# The impact of growth hormone (GH) on immunosenescence: exploring the role of B and T cells

**DOI:** 10.1007/s11102-025-01632-y

**Published:** 2026-01-12

**Authors:** Badra Bashir, Marcella van Hoolwerff, Fabian Benencia, Silvana Duran-Ortiz, Edward O. List, John J. Kopchick, Darlene E. Berryman

**Affiliations:** 1 Translational Biomedical Sciences Doctoral Program, Ohio University, Athens, OH 45701 USA; 2 Institute of Molecular Medicine and Aging, Heritage College of Osteopathic Medicine, Ohio University, Athens, OH 45701 USA; 3 Department of Biomedical Sciences, Heritage College of Osteopathic Medicine, Athens, OH 45701 USA; 4 Diabetes Institute, Ohio University, Athens, OH 45701 USA; 5 Office of Research and Grants, Medical Education Center 222, Heritage College of Osteopathic Medicine, Ohio University, Athens, OH 45701 USA

**Keywords:** Immunosenescence, Growth hormone, GHR-/- mice, B cells, Aging-associated b cells (ABC), T cells, Aging

## Abstract

**Purpose:**

Immunosenescence is a gradual decline in immune function, leading to increased susceptibility to infections and autoimmune conditions. Growth hormone (GH) has been shown to have an effect on both immune function and aging. In fact, the absence of GH-induced intracellular signaling can slow the aging process, as demonstrated by the longest-lived laboratory mouse (GH receptor gene disrupted or GHR-/- mice). Because GH receptors (GHR) are expressed in B and T cells, and these cells undergo age-related changes that impact immune function, we hypothesized that decreased GH action protects from immunosenescence. To validate this hypothesis, this study aimed to characterize differences in B cell and T cell populations within the lymphoid organs of aged female GHR-/- mice (24 months of age) compared to wild-type controls.

**Methods:**

B and T cell populations in mouse blood, spleen, thymus, and bone marrow (BM) were analyzed by multicolor flow cytometry.

**Results:**

The current study showed significantly higher levels of anti-inflammatory follicular (FO) B cells in spleens and BM and lower levels of pro-inflammatory aging-associated B cells (ABC) in the spleens, BM, and blood of aged GHR-/- mice compared to WT mice. In addition, T cell populations in aged GHR-/- mice showed higher levels of naïve T cells and lower levels of memory T cells in the thymus, BM, spleen, and blood.

**Conclusion:**

Female GHR-/- mice are protected from age-related shifts in lymphocyte populations, suggesting that the absence of GH action mitigates immunosenescence. These results offer novel insights into mechanisms and therapeutic strategies to preserve immune balance and combat age-related immune dysfunction.

**Supplementary Information:**

The online version contains supplementary material available at 10.1007/s11102-025-01632-y.

## Introduction

Aging is a complex and gradual pathophysiological process that is linked to a decline in both physical and mental function in humans [1]. Unfortunately, old age is the greatest risk factor associated with many diseases, including metabolic diseases, cardiovascular disorders, neurodegenerative diseases, neoplasia, and autoimmune diseases [2]. One key contributor to the detrimental effects of aging is immunosenescence, characterized by the decline in immune function with age [3, 4], vulnerability to infections due to dampened immune response with aging [5], chronic inflammation associated with metabolic diseases, and autoimmune conditions [6]. Immunosenescence increases the risk and severity of diseases and thus plays a pivotal role in the morbidity and mortality of the elderly population [7]. Given the aging global population and the emerging threat of infectious diseases [8], exploring mechanisms that can halt the immunological clock and protect against immunosenescence is crucial.

Immunosenescence affects both adaptive and innate immune responses, leading to notable changes in their function. Age-related changes in hematopoietic stem cells [9] include decreased lymphopoiesis, resulting in lower B- and T-cell production rates [10]. Bias in bone marrow (BM) progenitor pools leads to a reduction in differentiation intermediates in primary and secondary lymphoid organs. Despite this biased potential, mature B and T cell numbers remain stable [11, 12]; however, changes in specific cell subsets, clonal composition, and diversity become more pronounced with age. While the mechanisms behind these significant functional changes remain unclear, they likely involve age-related alterations in both developing and mature B- and T-cell compartments [13, 14]. Indeed, aging is associated with shifts in the proportions of T-cell subsets, including increased frequency and number of regulatory T cells [15], reduction in generation of functional memory CD4 + T cells [16], and memory-like (CD44hi) CD8 + T cells [17]. Recently, a novel subset of mature B cells was discovered that accumulates with age. These age-associated B cells (ABC) are defined as a B cell subset with unique phenotypic and transcriptional regulators usually determined by the presence of select cell surface markers (CD11c on B220 + CD19 + CD21- CD23- and T-bet+) and accumulate with age and correlate with immunosenescence [18]. Splenic ABC continually increase in the number and proportion of mature B cells with increasing age. ABC increase at the expense of another subset of B-cells called Follicular B cells (FO) [19]. These naturally occurring ABC present at low frequency at 3 to 6 months of age in mice, increase to 30% by 18 to 22 months, and expand to 50% of splenic B cells by the age of 24 to 30 months [14, 19, 20]. Collectively, these age-related changes to B- and T-cell subsets contribute to reduced functionality, diminished diversity, and impaired adaptive immunity, all contributing to immunosenescence.

The GH receptor gene-disrupted (GHR-/-) mouse is the current titleholder of the Methuselah Mouse Prize (for the world’s longest lived mouse), with one mouse dying just a week short of his fifth birthday, the equivalent of a human lifespan of 150 years [21, 22]. These mice are also resistant to age-related chronic diseases such as diabetes and cancer; however, the underlying mechanism influencing both the health span and lifespan in GHR-/- mice remains an area of active research with many important questions remaining. For example, does GH also play a role in immune aging? While this is not known, GH has been linked to other aspects of immune cell function. For example, GH has been shown to play a role in lymphopoiesis and thymopoiesis [23]. GHR has higher expression levels in B cells as compared to T cells and neutrophils [24, 25], suggesting that B cells are likely the most responsive to GH action among immune cell types. GH has been shown to play an important role in B and T cell differentiation, maturation, and proliferation by regulating the homeostasis of pro- and anti-inflammatory cytokines and chemokines [26]. GH also increases in vitro levels of IgG, IgE, IgM, and IgA antibodies from B cells [27].

Thus, in this study, aged GHR-/- mice (completely devoid of GH action) were used to explore immune cell subsets in blood, BM, thymus, and spleen. Follicular (FO) B cells, age-associated B cells (ABC), marginal zone (MZ) B cells, and Memory (IgM-/+) B cells, as well as Naïve T and memory T cells, were quantified. Our findings show that B and T cell subsets in older (20–24-month) GHR-/- female mice are less susceptible to the age-related shifts in lymphocyte populations commonly observed in WT mice.

## Materials and methods

### Mice

Female GHR-/- mice in a C57BL/6J background and littermate WT controls at 20–24 months of age (*n* = 6/genotype) were used as the aged cohort. Mice were bred and maintained at Ohio University. Mice were housed at 22 (± 2) °C with a 14-hour light and 10-hour dark cycle and with 2 to 4 mice per cage. For the entire lifespan, mice had *ad libitum* access to standard laboratory rodent chow (Prolab RMH 3000 3000, 26% protein, 14% fat, 60% carbohydrates). All procedures were approved by Ohio University’s Institutional Animal Care and Use Committee.

### Body composition

Body weight and body composition were measured using Bruker Minispec ND2506. Measurements were taken the day before dissection. Body composition measurements of fat, free body fluid, and lean tissue were recorded as previously described [28].

### Tissue collection

All mice were fasted for 12 h prior to dissection, and mouse weights were recorded. Mice were anesthetized with CO_2_, followed by immediate blood collection in heparin-coated tubes (Microvette CB 300 LH, Sarstedt) by retro-orbital bleeding. After collecting blood, the mice were sacrificed by cervical dislocation, followed by collection of spleen, thymus, and one femur BM.

### Single-cell suspension preparation

The spleen and thymus were weighed and placed in Krebs-Henseleit Buffer solution (KHB, 11 mM D-glucose, 1.2 mM MgSO_4_, 1.2 mM KH_2_PO_4_, 4.7 mMKCl, 118 mMNaCl, 2.5 mM CaCl_2_, 25 mM NaHCO_3_, 0.5% (w/v) BSA, pH 7.4, Sigma), minced and filtered through 100 µ Falcon cell strainers (Fisher Scientific). The femur was cleaned of skin, fat, and muscle; BM was extracted from the femur by cutting it in half and flushing it with KHB solution. The suspension was then filtered through a 100 μm strainer. The spleen and BM samples were then centrifuged for 5 min at 400 x g at 4 °C. Hereafter, the spleen and BM cell suspension and the collected blood were subjected to red blood cell (RBC) lysis by adding 5 ml of ACK lysis buffer (Thermo Fisher Scientific) to the pellets and incubating at room temperature for 5 min. The thymus cell suspension was washed with KHB solution. All samples were then centrifuged for 5 min at 400 x g at 4 °C, and the pellet was suspended in FACS buffer (2% FBS, 0.05% sodium azide in PBS).

### Flow cytometric analysis of cell-surface antibodies

The cells were counted using Countess automated cell counter (Invitrogen) by Trypan Blue staining; then, the spleen samples were diluted in FACS buffer to stay within the measurement range (1–2 × 10^6^). Cells were resuspended at 1–2 × 10^6^ cells/ml for each sample in FACS blocking buffer (FACS buffer with 10% horse serum) to prevent non-specific binding. B-cells were stained with fluorophore-conjugated monoclonal antibodies. For B cells from spleen, blood, and BM the following antibodies were used: CD45-APC-Cy7 (30-F11; BD Pharmingen); CD86-AF700 (GL-1; BioLegend), CD73-PerCP (eBioTY/11.8), B220-PeCy7 (RA3-6B2), CD93-APC (AA4.1, all from eBioscience), IgM-PE (eB121-15F9), CD21-FITC (eBio4E3), and CD23-biotin (B3B4). The following cell surface markers were used to identify age-associated B-cells (CD86 + CD73 + B220 + CD93/CD43-CD21-CD23-), Marginal Zone B cells (MZ, B220 + AA4.1/CD43-CD21 + CD23-), Follicular B cells (B220 + AA4.1/CD43-CD21-CD23+), and Memory B cells (CD45 + B220 + IgM+/-CD73+). Negative selection was done during staining to exclude immature B cells (e.g., AA4.1/CD93 is a marker for transitional B cells, and CD43 is a marker for B1 B cells). The following fluorophore-conjugated monoclonal antibodies were used for T cell staining: CD4-AF488 (GK1.5), CD3-PE (145-2C11), CD44-biotin (IM7; all from eBioscience), CD8-PeCy7 (53 − 6.7; BioLegend), CD45-APC (30-F11), and CD62L-APC-Cy7 (MEL-14), both from BD Pharmingen. For each tissue, isotype controls were prepared to be able to detect background staining. These isotypes were: FITC mouse IgG2b κ (27–35), AF700 mouse IgG1 κ (MOPC-21), APC-Cy7 rat IgG2a (B39-4; all from BD Biosciences), PE mouse IgG2b (eBMG2b), PeCy7 rat IgG2a κ (eBRG1), APC rat IgG1 κ (eBRG1; all from eBioscience), PerCP hamster IgG (HTK888; BioLegend) (supplementary material Table 1). Secondary stains were PE-Texas Red (BD Pharmingen) or QDot705-coupled streptavidin (Invitrogen) used to detect biotinylated primary antibodies (supplementary material Table 2). To the samples with biotinylated antibodies, streptavidin PE-Texas Red (BD Pharmingen) was added in a 1:500 dilution.

Multicolor flow cytometry was performed on a FACSAria II flow cytometer (BD Biosciences) with a blue (488 nm) and red (633 nm) laser (supplementary material Table 3) using FACSDiva 8.0.1 software (BD Biosciences), where 10,000 events were collected per sample. The data were calculated by FACSAria II with 4-log scale axes and further analyzed using FlowJo 10.1 analysis software.

#### Cell counting

For flow cytometric counting, splenocytes cleared of RBCs (adding 5 ml of ACK lysis buffer (Thermo Fisher Scientific) to the pellets and incubating at room temperature for 5 min) were stained with the antibodies mentioned above. Singlet leukocytes were counted using BD Biosciences FACSDiva software. Cell frequency and number were counted according to the magnetic field and attraction of cell surface markers.

#### Statistical analysis

Statistical analyses were performed using GraphPad Prism 8, and mean comparisons were performed by unpaired Student’s t-test (two-tailed) for body weight, body composition, and multiple T and B cell subsets of two genotypes. Data are expressed as mean ± standard deviation (SD). Significant differences were considered at *P* < 0.05.

## Results

### Body composition and weight differences of aged GHR-/- mice as compared to age-matched WT mice

**Fig. 1:**
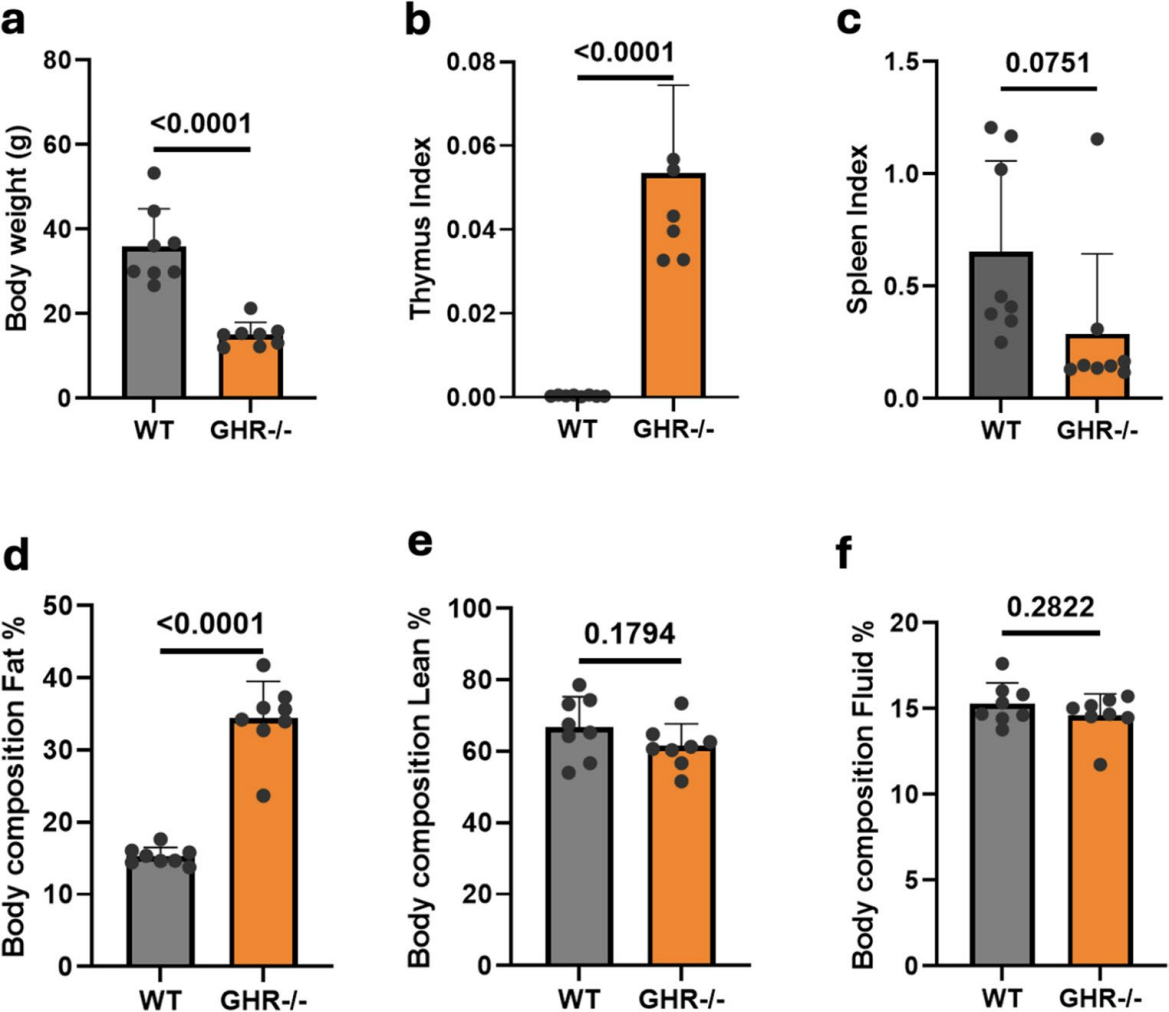
Comparison of body weight, tissue weight, and body composition of female WT and GHR-/- mice at 20–24 months of age. (**a**) Total body weight for each genotype (**b**) thymus weights as a percentage of body weight (**c**) spleen weights as a percentage of body weight. Body composition expressed as a percent of body weight (**d**) fat % (**e**) lean % (**f**) fluid %. Data are expressed as mean ± SD, *n* = 6 to 8/group

Aged GHR-/- mice had significantly lower body weight (42% of the age-matched WT controls) (Fig. 1a). The absolute weight of thymus in GHR-/- mice (0.008 g ± 0.001) was smaller than that of WT (0.01 g ± 0.001) controls. However, when normalized the tissue weight to body weight, GHR-/- mice had a significantly larger (*P* < 0.0001) thymus as compared to controls (Fig. 1b). For the spleen, no significant difference in weight was observed although there was a large standard deviation, and GHR-/- spleens tended to be smaller as compared to age-matched controls (Fig. 1c). As expected, GHR-/- mice had a significant increase in percentage of fat mass (34.4% in GHR-/- mice and 15.3% in WT mice) although there were no significant differences in percentage of fluid or lean mass (Fig. 1d-f). The body length of the GHR-/- mice was two-thirds of the age-matched WT mice (7.3 cm ± 0.27 for GHR-/- mice and 10.6 cm ± 0.10 for WT mice).

### Splenic mature B cell subsets from aged GHR-/- mice as compared to WT mice

**Fig. 2:**
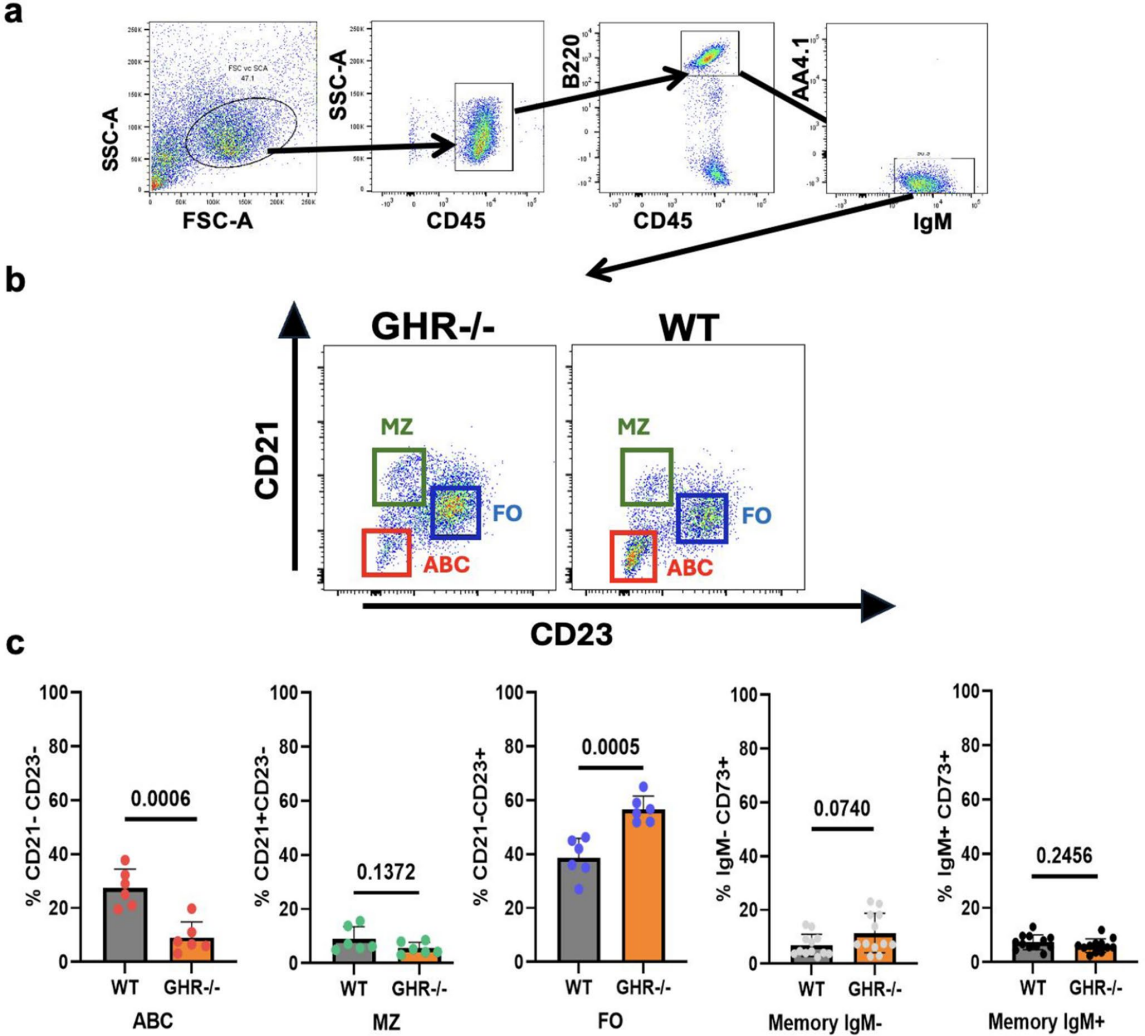
Composition of the splenic B cell pool of old, age-matched GHR-/- and WT mice. The spleens of 20 to 24-month-old female GHR-/- and WT mice (*n* = 6/genotype) were stained to evaluate percentages of the major B cell subsets by flow cytometry. (**a**) The gating strategy included gating cells first by CD45 + and then by B220 + AA4.1-IgM + cells to exclude transitional (AA4.1+) B cells. (**b**) A representative dot plot of splenic B cells from a single WT and GHR-/- spleen. (**c**) Frequencies of ABC, MZ, FO, IgM-/+ memory B cells B cells, in the full cohort of WT and GHR-/- mice (gating strategy shown in supplementary Fig. 3a). Data are expressed as mean ± SD, *n* = 6 to 8

We compared the splenic B-cell pools by measuring the cell surface phenotypes of aged female GHR-/- and WT mice. Using flow cytometry to quantify major B cell subsets, the gating strategy was to first generate a CD45 + leukocyte gate, and then B220 + expression was used to define the B cell population. IgM was used as a marker of immature B cells, and AA4.1 expression was used to exclude transitional (AA4.1+) B cells, as shown in Fig. 2a. Age-associated B cells (ABC - B220 + CD93/CD43-CD21-CD23-), Marginal Zone B cells (MZ - B220 + AA4.1/CD43-CD21 + CD23-), Follicular B cells (FO - B220 + AA4.1/CD43-CD21-CD23+) and Memory B cells (CD45 + B220 + IgM+/-CD73+) populations were compared between GHR-/- and WT mice (Fig. 2b). Frequencies of splenic FO, ABC, MZ, and Memory B cells are shown in Fig. 2c. GHR-/- mice have significantly lower frequency and number of ABC cells and significantly higher frequency of the FO population as compared to age-matched WT control mice. No significant changes were observed in the MZ B cell subset. The frequency and number of FO in GHR-/- mice decreased by 1.5-fold (57% in GHR-/- to 38% in WT mice at 20–24 months of age) while the ABC increased by 4-fold (9% in GHR-/- to 27% in WT mice), mimicking a younger immune profile for GHR-/- mice in terms of the B cell subsets compartment in the spleen. No differences in memory B cells (gating strategy shown in supplementary Fig. 3a), defined as IgM- Memory B cell: CD45 + B220 + IgM- CD73 + and as IgM + Memory B cell: CD45 + B220 + IgM + CD73 + were observed between the GHR-/- and controls [29].

**BM and blood B cells subsets from old GHR-/- mice as compared to age-matched WT controls**

**Fig. 3:**
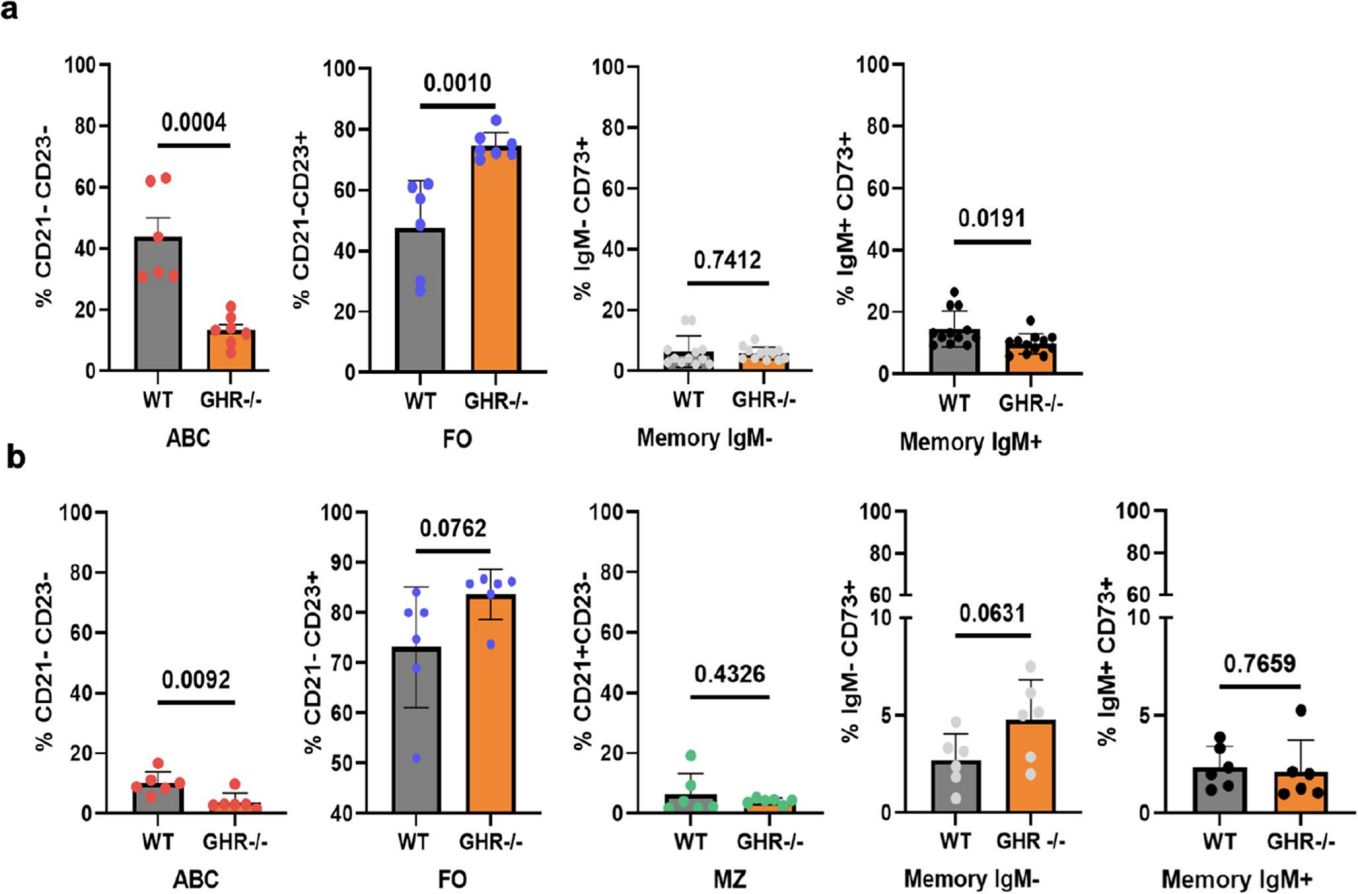
Composition of the B cell pool of old, age-matched GHR-/- and WT mice. BM and blood of 21 to 24-month-old female GHR-/- (*n* = 7) and WT mice (*n* = 6) were stained to evaluate percentages of the major B cell subsets by flow cytometry. (**a**) Frequencies of BM ABC, FO, IgM-/+ memory B cells populations (gating strategy shown in supplementary Figs. 1 and 3b). (**b**) Frequencies of blood ABC, FO, IgM-/+ memory B cells populations (gating strategy shown in supplementary Figs. 2 and 3c). Data are expressed as mean ± SD, *n* = 6 to 8/group

Comparisons of the BM and blood B cell pools between aged female WT and GHR-/- mice (*n* = 6) were made using a similar gating strategy as described above for spleen B cell subsets shown in supplementary Figs. 1 and 2. As expected, we were not able to detect a relevant population of MZ B cells in BM, which are typically present in the spleen and blood, but ABC, FO, and Memory B cells were identified. As depicted in Fig. 3a, GHR-/- mice showed a significant increase in anti-inflammatory follicular (FO) B cells and decreased pro-inflammatory aging-associated B cells (ABC) in BM, similar to the results observed in the spleen. In addition, we were able to detect lower levels of memory B cells, IgM + CD73+, in BMs of GHR-/- mice compared to controls Fig. 3a (gating strategy shown in supplementary Fig. 3b and c). Typically, in aging, memory B cells accumulate in BM while there is a reduction in naïve B cells. Consistent with the previous finding in other tissues, blood showed a significant reduction in ABC in GHR-/- mice compared to controls. While FO B cells showed an increasing trend (Fig. 3b), the difference did not reach significance in the blood of GHR-/- mice relative to controls.

### T-cell subsets in spleen and BM in old GHR-/- mice as compared to age-matched WT mice

**Fig. 4:**
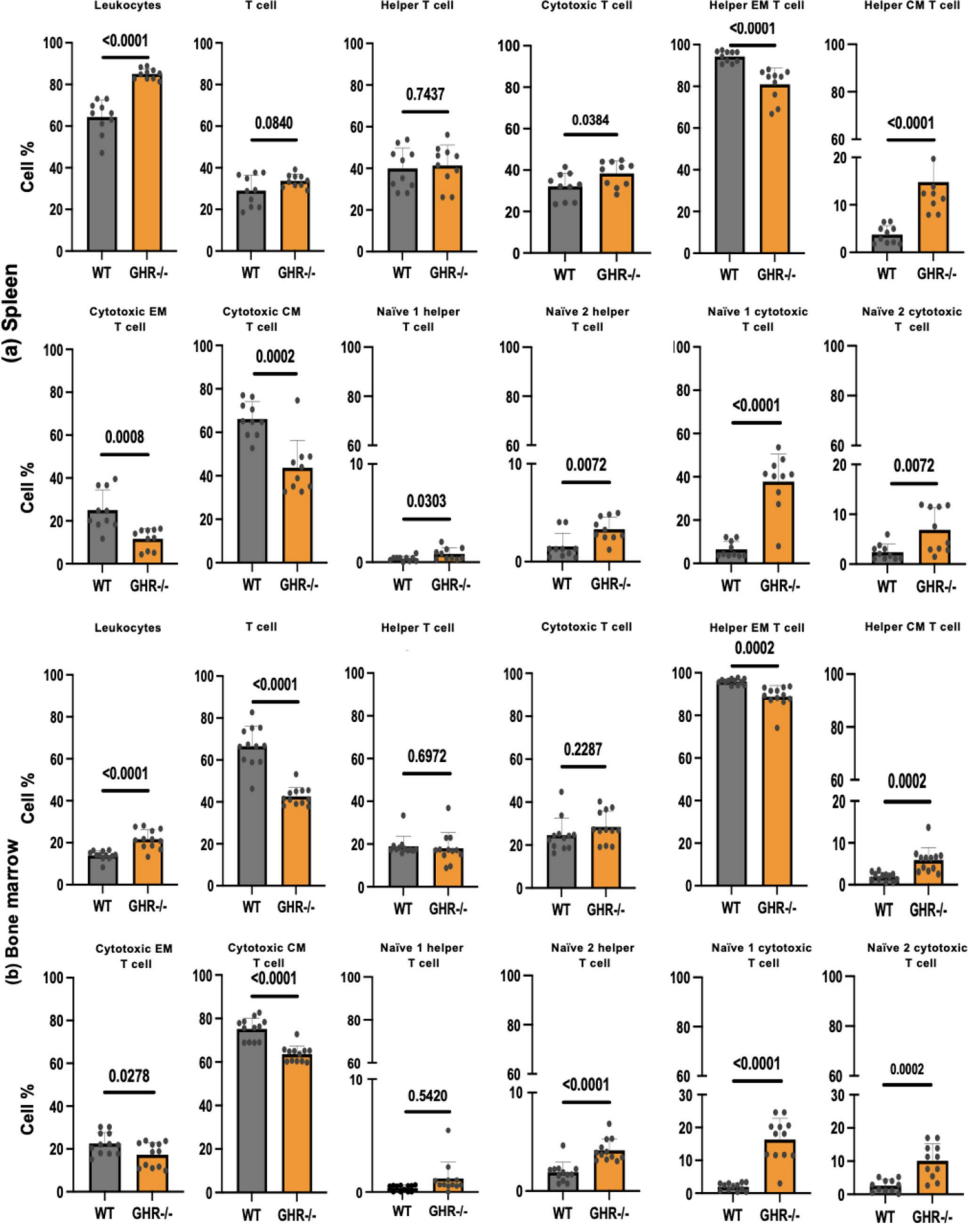
Analysis of the spleen and BM T cell pool of old, age-matched GHR-/- and WT mice. Leukocytes (CD45^+^), T cells (CD45^+^ CD3^+^), Helper T cells (CD4^+^), Cytotoxic T cells (CD8^+^), Effector memory helper T cells (EM; CD4^+^ CD44^+^CD62L^−^), Central memory helper T cells (CM; CD4^+^ CD44^+^, CD62L^+^), Effector memory cytotoxic T cells (EM; CD8^+^ CD44^+^CD62L^−^), Central memory cytotoxic T cells (CM; CD8^+^ CD44^+^, CD62L^+^), Naïve 1 helper T cells (CD4^+^CD44^−^CD62L^+^), Naïve 2 helper T cells (CD4^+^CD44^−^CD62L^−^), Naïve 1 cytotoxic T cells (CD8^+^CD44^−^CD62L^+^); and Naïve 2 cytotoxic T cells (CD8^+^CD44^−^CD62L^−^) in the full set of WT and GHR-/- mice. (**a**) Frequencies of the spleen T cell pool of old, age-matched GHR-/- and WT mice. (**b**) Frequencies of the BM T cell pool of old, age-matched GHR-/- and WT mice. The subpopulations are normalized to the parental population depicted under the lines. Data are expressed as mean ± SD, *n* = 8 to 10/group

**Fig. 5:**
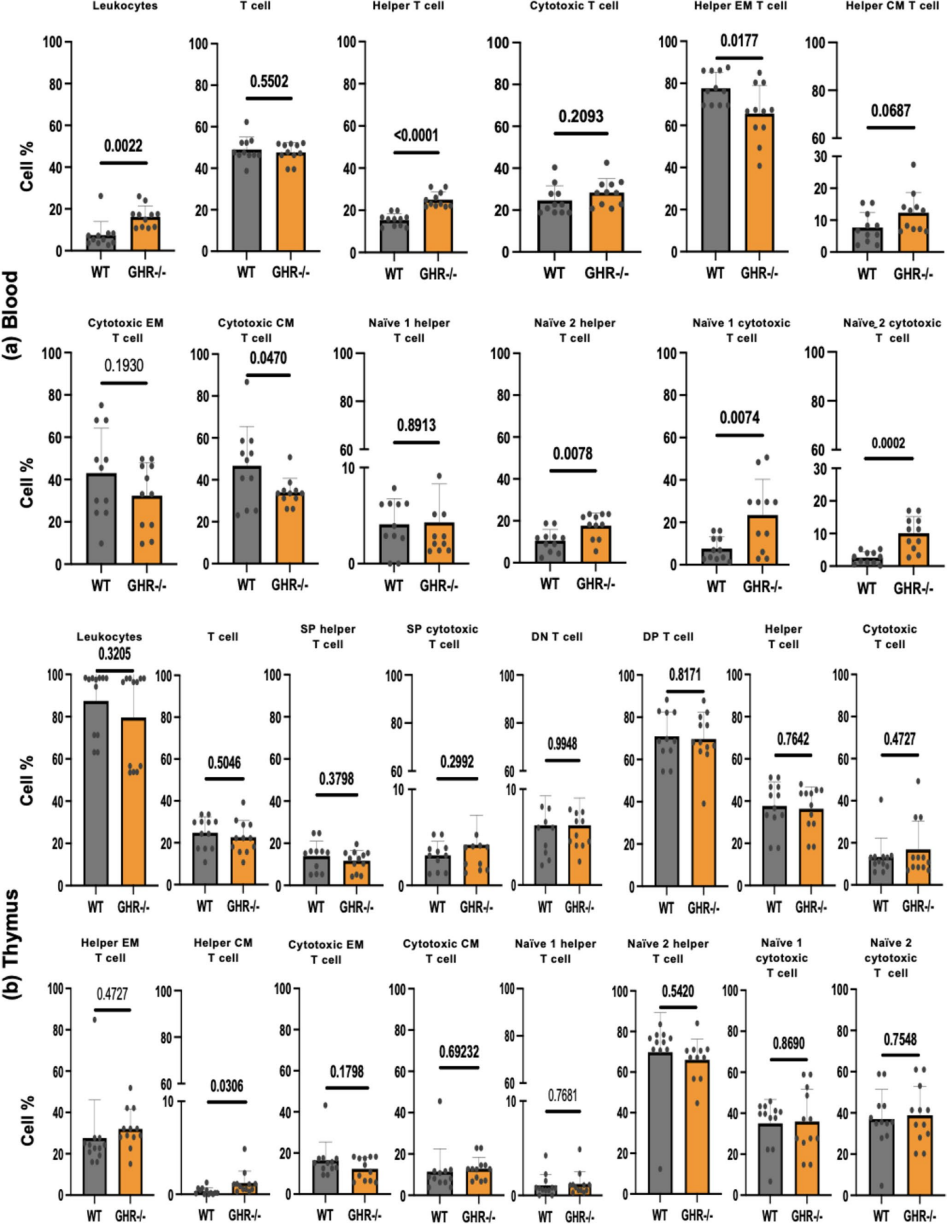
Composition of the T cell pool of old, age-matched GHR-/- and WT mice in blood and thymus Leukocytes (CD45^+^), T cells (CD45^+^ CD3^+^), Helper T cells (CD4^+^), Cytotoxic T cells (CD8^+^), Effector memory helper T cells (EM; CD4^+^ CD44^+^CD62L^−^), Central memory helper T cells (CM; CD4^+^ CD44^+^, CD62L^+^), Effector memory cytotoxic T cells (EM; CD8^+^ CD44^+^CD62L^−^), Central memory cytotoxic T cells (CM; CD8^+^ CD44^+^, CD62L^+^), Naïve 1 helper T cells (CD4^+^CD44^−^CD62L^+^), Naïve 2 helper T cells (CD4^+^CD44^−^CD62L^−^), Naïve 1 cytotoxic T cells (CD8^+^CD44^−^CD62L^+^); and Naïve 2 cytotoxic T cells (CD8^+^CD44^−^CD62L^−^) in the full set of WT and GHR-/- mice. (**a**) Frequencies of the blood T cell pool of old, age-matched GHR-/- and WT mice. (**b**) Frequencies of the thymus T cell pool of old, age-matched GHR-/- and WT mice. SP: single positive CD3 cells (CD8 or CD4); DP: double positive (CD4 + CD8+) T cells, DN: double negative (CD4- CD8-) T cells. Data are expressed as mean ± SD, *n* = 9 to 12/group

The gating strategy for distinguishing T cell subsets in the flow cytometric analysis is illustrated in Supplementary (S Fig. 4). Leukocytes were first gated based on CD45 expression. Further gating strategies for T cell (CD45^+^CD3^+^) populations, including helper CD4 + and cytotoxic CD8 + subsets, are depicted in S Fig. 4a. Naïve helper T cells were defined as N1: CD4^+^CD44^−^CD62L^+^, and N2: CD4^+^CD44^−^CD62L. Memory helper T cells were categorized as effector memory (EM): CD4^+^CD44^+^CD62L^−^ and central memory (CM): CD4^+^CD44^+^CD62L^+^ in S Fig. 5a. The N2 population has been rarely studied; however, we included it given its reported role in immunity in aged mice [30]. The gating strategies for all tissues (spleen, BM, blood, and thymus) were similar (depicted in S Fig. 5a, b and c, and 5d).

Overall, no significant differences were observed in the percentage of total splenic T cells (CD3+) or helper T cells (CD4+) in GHR-/- vs. WT (Fig. 4a). In contrast, BM of GHR-/- showed a significant decrease in the proportion of CD3 + T cells when normalized to the leukocyte population (Fig. 4b). Notably, cytotoxic CD8 + T cells were significantly increased in spleen, while no difference was observed in the GHR-/- mice BM compared to controls (Fig. 4a and b). Both spleen and BM of GHR-/- mice showed significantly reduced populations of memory T cells, including effector memory helper T cells (CD4 + EM) and cytotoxic memory T cells (CD8 + EM and CD8 + CM) (Fig. 4a and b). Interestingly with in the helper memory T cell compartment, central memory T cells (CD4 + CM) were higher in GHR-/- mice compared to WT controls (Fig. 4a). In contrast to the memory compartment naïve helper T cells (CD4 + Naïve 2) and naïve cytotoxic T cells (CD8 + Naïve 1 and 2), were significantly increased in both spleen and BM relative to controls (Fig. 4a and b). The frequency and number of naïve T cells in GHR-/- mice increased while the memory T cells decreased, mimicking the younger immune profile in terms of the T cell subsets compartment in the spleen.

### Specific T-cell subsets blood and thymus in old GHR-/- mice as compared to age-matched WT mice

An analysis of white blood cells revealed that GHR-/- mice exhibited a significant increase in the overall helper CD4 + T cell population within leukocytes. However, no significant differences were observed in the proportions of T cells (CD3+) and cytotoxic (CD8+) T cell subsets between genotypes (Fig. 5a). Interestingly, the memory helper (CD4^+^EM; effector memory) and cytotoxic (CD8^+^CM; central memory) T cell compartments were significantly decreased in the GHR-/- mice (Fig. 5a). In contrast, the naïve helper T cell population (CD4^+^ naïve 2) showed a significant increase in the GHR-/- mice compared to the WT controls, and the same trend was visible in the naïve cytotoxic T cells (CD8^+^ naïve 1 and naïve 2) (Fig. 5a).

In the thymus, overall proportions of T cell subsets remained largely unchanged between genotypes (Fig. 5b). While examining specific subsets, no differences were detected in CD4 + T and CD8 + T cells. Notably, central memory helper T cells (CD4 + CM) were elevated in GHR-/- mice. Differences were observed in the cytotoxic T cell compartment, where effector memory (CD8 + EM) and central memory (CD8 + CM) T cells showed a decreasing trend in GHR-/- mice but did not reach statistical significance. Conversely, naïve cytotoxic T cells (CD8 + Naïve 1) were increased in the absence of GH signaling.

## Discussion

This study is the first to examine how lifelong deficiency of GH action influences immune cell subsets, specific B and T cell subsets, in the context of immunosenescence. More specifically, GHR-/- mice and WT controls at 20–24 months of age were utilized to compare the distribution of T and B lymphocyte populations in primary and secondary lymphoid organs and peripheral blood. Our findings showed lower levels of B and T cell populations associated with aging and higher levels of B and T cell naïve populations in old GHR-/- mice compared to age-matched controls. Additionally, GHR-/- mice showed a higher relative thymic mass respect to controls. It is noteworthy to highlight that the thymus is an organ that regresses with aging, which determines a decrease in the output of new T cell populations. Based on previous studies [14–19], these collective results suggest that GHR-/- mice have a younger immune profile compared to their chronologically age-matched WT controls and maintain immune cell homeostasis even in older age, which may help mitigate the detrimental effects of immunosenescence and inflammaging in these mice.

GHR-/- mice exhibited intriguing alterations in B lymphocyte subsets in BM and spleen, showing lower levels of the inflammatory ABC population than age-matched controls. It has been previously reported that pituitary/thyroid hormones can affect the B cell compartment. For example, B cell lymphopenia has been shown in other longevity mouse models harboring defects in the pituitary/thyroid axis, e.g., Snell, Ames, and *lit/lit* mice, thus highlighting the complexity of the immune regulation in aging [31]. Emerging evidence strongly suggests that GH and IGF-1 act as positive regulators of B-cell lymphopoiesis [32, 33]. However, the role of GH in specific subsets of B cells has not been reported. B cells, key players in the adaptive immune system, exhibit alterations in their subsets with advancing age. Among these, age-associated B cells (ABC) represent a novel subset identified about a decade ago by Hao et al. and defined by surface markers CD21/CD35-CD23-; phenotypically distinct from other B-cell populations [14]. More recently, a similar gating strategy has been consistently applied by Frasca et al. [18], and was therefore used in the present study to enable direct comparison with existing literature. However, in future studies including additional markers such as T-bet or CD11c may further refine the specificity and functional characterization of this population [34]. ABC accumulate gradually with age and compete homeostatically with FO B cells and MZ B cells [18, 20]. ABC produce cytokines (IL-4 and IL-10) via innate receptor stimulation and skew T cells toward a TH17 fate at the expense of other T helper cell subsets [14]. Thus, ABC not only drive immune homeostasis toward a senescent fate [35] but also indirectly promote the activation and concentration of cytotoxic T cells [36–38]. Therefore, reduction in GH action not only reduces the accumulation of senescent ABC but at the same time increases the naive FO B cells (both in spleen and BM) that can provide a robust and long-lasting humoral immune response [39].

In our studies, analysis of peripheral blood samples showed a significant reduction in ABC in GHR-/- mice compared with controls. However, unlike observation of the spleen and BM, the difference in FO B cells did not reach significance. Notably, a previous study reported a significant increase in the percentage of ABC in the peripheral blood of 24-month C57BL/6 mice compared to young controls [40], although Hao at el [14]. questioned this finding and reported variability in the abundance of ABC in peripheral blood. Although previous studies on GH-releasing hormone knock-out mice (Ghrh^−/−^) mice report a reduced frequency of total B cells and an increase in the proportion of T cells in the blood, they did not assess specific B cell subsets [41]. A possible explanation is the reported variability of the frequency of cells in peripheral blood at different time points in the same mice [19]. Our data also demonstrate a disconnect between splenic and blood B cell subsets, which raises an important caution for studies that only examine B cell subsets in peripheral blood, as the levels in the spleen are not mirrored in the blood.

It is important to note that biological sex can influence the immune profile, with males and females often exhibiting distinct immune responses due to differences in sex hormones, genetics, and immune regulation [6, 42]. These sex-based differences may impact susceptibility to disease, vaccine responses, and overall immune aging. GHR-/- female mice showed a significant reduction in ABC accumulation at 20–24 months compared to WT controls; however, other studies have shown that WT female mice have greater ABC accumulation as compared to WT males at both younger and older age points [43]. The accumulation of ABC is associated with autoimmune diseases, which aligns with the observation that nearly 80% of such conditions occur in females [43–45]. ABCs proliferate vigorously in response to stimuli that activate the endosomal nucleic acid-sensing Toll-like receptors TLR7 or TLR9 [19]. The presence of TLR7 on the X chromosome is thought to contribute to the female bias in ABC appearance [46]. Likewise, GH action in mice is sexually dimorphic, with males typically exhibiting pulsatile GH secretion patterns that drive stronger hepatic STAT5 signaling and downstream gene expression, whereas females show more continuous GH secretion, leading to differential regulation of GH-responsive genes [47]. This study, which only included female mice, could provide critical insights into the mechanisms underlying ABC activation and the progression of autoimmunity, particularly in the context of GH signaling pathways.

GHR⁻/⁻ mice showed increased adiposity, and prior studies have demonstrated depot-specific changes in adipose tissue deposition [48]. Notably, despite the increased fat mass, GHR-/- mice show features of “healthy obesity”, including elevated circulating adiponectin levels [49], which are thought to contribute to their metabolically protective phenotype. Interestingly showed no significant difference in spleen weight but had a significant (*P* < 0.0001) increase in thymic weight when adjusted for total body weight. Previous studies demonstrate that GH-deficient lit/lit mice and pituitary dwarf mice (Snell and Ames) have marked spleen atrophy and B-cell lymphopenia (diminished B cell frequency in the spleen, lymph nodes, and blood) [42, 49, 50]. Consistent with these findings, spleen weight (% of BW) in GHR⁻/⁻ mice tended to be smaller compared to age-matched controls, although this difference did not reach statistical significance.

In contrast to the spleen, thymic changes in GHR⁻/⁻ mice appear distinct from other GH/IGF-deficient models. Snell and Ames mice show early thymic involution and reduced primary immune responses, whereas Ghrh⁻/⁻ mice, another model of severe GH and IGF-1 deficiency, do not present obvious immunodeficiency or thymic atrophy at least at 18 months of age [42]. Interestingly, our study demonstrates delayed thymic involution in aged GHR⁻/⁻ mice relative to controls, as indicated by thymus weight (% of BW). This observation is consistent with findings in PAPPA⁻/⁻ mice, which also exhibit delayed thymic aging despite normal systemic GH/IGF levels, due to reduced IGF signaling [51]. More specifically, in PAPPA⁻/⁻ mice, where local IGF bioavailability is reduced in the thymus despite normal systemic IGF-1 and GH, lower thymic IGF-1/IGF-1R expression and increased IGFBP4 are associated with preserved cortical–medullary architecture, maintenance of immature double negative and double positive thymocyte pools, higher thymic and splenic T-cell Receptor Excision Circles content, sustained bone-marrow thymus-seeding progenitors, and a larger, more diverse naïve T-cell compartment into old age, indicating that chronic attenuation of thymic IGF signaling slows exhaustion of progenitors and delays age-related thymic involution. By analogy, we speculate that in GHR⁻/⁻ mice, reduced GH/IGF-1 signaling in thymic epithelial and stromal cells lowers proliferative and metabolic stress, leading to delayed or preserved thymic involution. Supporting this, flow cytometric analysis of thymic T-cell subsets in GHR⁻/⁻ mice revealed a non-significant trend toward increased helper T cells with a concomitant decrease in memory cytotoxic cells. The frequency of total leukocytes and double-positive thymocytes showed no significant difference, contrasting with the substantial declines seen in other long-lived models such as Snell dwarfs [52].

Thymic involution, a hallmark of immune aging, begins early, around 6 weeks in mice and roughly one year in humans, much earlier than most other organs show signs of aging [45]. Thymic involution due to aging is a well-known characteristic of the aging immune system and is thought to play a substantial role in immunosenescence [50]. Preservation of age-associated thymic atrophy in GHR-/- mice might be responsible for the continuous production of naïve T cells, and maintenance of lymphoid-to-myeloid homeostasis to mount an effective immune response. This finding reinforces the notion of a slower aging and a more youthful immune system in GHR-/- mice at an older age, which helps to prevent the deterioration of the adaptive immune response and activation of autoreactive T cells [51]. Further experiments assessing the expression of senescence markers, the functional capacity, and proliferative potential of T cell subsets derived from aged GHR⁻/⁻ mice would help clarify the broader implications for immune resilience.

Our study is the first to demonstrate that at advanced ages (20–24 months), female GHR⁻/⁻ mice exhibit a significant increase in the frequency of naïve T cell subsets and a corresponding reduction in memory T cell subsets, compared to age-matched WT controls. Although previous studies in Ghrh⁻/⁻ mice reported a similar trend, the observed differences did not reach statistical significance at older age points (18 months) in their study [41]. Age-related helper and cytotoxic T cell composition changes are delayed in GHR-/- mice, which is comparable to the immune cell composition of younger mice [52, 53]. Thus, GHR-/- mice at older ages show younger T cell populations, and naive cells could differentiate and proliferate in response to antigens, potentially enhancing immune responses in aged GHR-/- mice. These findings support the idea of preserved immune cells, better defense against infectious agents, and reduced immunosenescence. To that end, two groups previously demonstrated that adipose tissue [54] and T cells [55] are protected against immunosenescence in GHR-/- mice. Our lab previously compared accumulation of adipose-tissue macrophages and other stromal vascular fraction (SVF) leukocytes across multiple white adipose depots in GHR-/- mice at young (8-month) and old (24-month) mice [54]. At 8 months of age, GHR-/- mice demonstrated modest, depot-specific changes in T cells but no significant changes in macrophages. Serum cytokine levels at 8 months were unchanged, except for an increase in G-CSF in GHR-/-serum. At 24 months, there was no change in T cells, T helper cells, or M1 or M2 macrophages, while cytotoxic T cells in subcutaneous adipose tissue showed a significant reduction compared to controls. In addition, there was no difference between GHR-/- and controls in the serum levels of IL-6, MCP-1, or TNF in this study, although other studies have reported that GHR-/- mice have elevated circulating adiponectin [56]. As adipose tissue T-cell and macrophage numbers, as well as circulating cytokines, are not dramatically altered in GHR⁻/⁻ mice, this suggests that splenic and blood phenotypes are not simply a direct reflection of immune cell sequestration in adipose depots. However, we did not directly phenotype innate immune subsets in spleen, thymus, or blood in the current work. Our goal was specifically to capture late-life immunosenescence; therefore, we focused on 24-month-old females.

Taken together, our findings suggest that immunosenescence is attenuated in GHR-/- mice, with less severe alterations in B and T cell subsets. Accordingly, reduced GH signaling is associated with a decrease in pro-inflammatory cytokines [57]. Persistent low-grade inflammation is known to impair the ability of immune cells to effectively respond to antigens. However, the reduced inflammatory microenvironment may enhance immune sensitivity to antigens by limiting exposure to pro-inflammatory cytokines and preventing T-cell exhaustion [58]. This suggests a potentially superior immune response in these mice. This study did not assess functional phenotypes of sorted immune cells or attempt to distinguish cell-intrinsic from systemic effects due to global GHR gene deletion; thus, our data cannot determine whether the observed immune changes reflect direct GH action on immune cells or indirect systemic changes. Addressing this distinction remains an important next step. Future studies comparing the immune responses of GHR⁻/⁻ mice and WT controls following antigen exposure could yield important insights into how altered GH signaling shapes immune function and resilience during aging. Further comparative analyses of immune cell populations across sexes and multiple ages of WT and GHR⁻/⁻ mice will be essential to determine whether the immune ‘age’ and phenotypic shifts reported here are conserved in males and in younger animals. Conversely, studies in bovine GH transgenic mice may illuminate how GH excess may accelerate immune aging, likely resulting in a decline in naïve lymphocytes and an expansion of memory and age-associated B and T cell populations. Overall, this study provides a foundation for defining targets to combat immunosenescence and autoimmune diseases via the GH axis and supports further investigation of GH antagonists like Pegvisomant as potential senolytic therapeutic agents.

## Supplementary information

Below is the link to the electronic supplementary material.

ESM 1 (DOCX 3.26 MB)

## Data Availability

No datasets were generated or analysed during the current study.
