# Supplementary material for: The impact of growth hormone (GH) on immunosenescence: exploring the role of B and T cells
Source: Pituitary. 2026 Jan 12;29(1):30. doi: 10.1007/s11102-025-01632-y (PMC12795870; doi:10.1007/s11102-025-01632-y)
Supplement: Supplementary file 1 — (DOCX 3.26 MB) [file 11102_2025_1632_MOESM1_ESM.docx]

**Supplementary Material**

**Table 1 | Overview stain I for T cell staining in blood, BM, spleen, and thymus**

|  | **AF488** | **PE** | **PE-Texas red** | **PeCy7** | **APC** | **APC-Cy7** |
| --- | --- | --- | --- | --- | --- | --- |
| Stain | CD3 | CD3 | CD44-biotin | CD8 | CD45 | CD62L |
| Isotype | Isotype | Isotype | Isotype-biotin | Isotype | Isotype | Isotype |
| FMO | Isotype | CD3 | Isotype-biotin | Isotype | CD45 | Isotype |

**Table 2 | Overview stain II for B cell staining in blood, BM, and spleen**

|  | **FITC** | **PE** | **PE-Texas red** | **PerCP** | **PeCy7** | **APC** | **APC-Cy7** | **AF700** |
| --- | --- | --- | --- | --- | --- | --- | --- | --- |
| Stain | CD21 | IgM | CD23-biotin | CD73 | B220 | AA4.1 | CD45 | CD86 |
| Isotype | Isotype | Isotype | Isotype-biotin | Isotype | Isotype | Isotype | Isotype | Isotype |
| FMO | Isotype | Isotype | Isotype-biotin | Isotype | B220 | Isotype | CD45 | Isotype |

**Table 3 | Properties of fluorophores using the FACS Diva with 2 lasers**

| **Laser** | **Fluorophore** | **Emission(nm)** | **Color** |
| --- | --- | --- | --- |
| Blue | Alexa Fluor 488 (AF-488) | 519 | Green |
| 488 nm | Fluorescein isothiocyanate (FITC) | 518 | Green |
|  | Phycoerythrin (PE) | 578 | Yellow |
|  | Phycoerythrin-Texas red (PE-Texas red) | 612 | Orange |
|  | Peridinin chlorophyll protein (PerCP) | 677 | Red |
|  | Phycoerythrin-Cyanine7 (PE-Cy7) | 785 | Infrared |
| Red | Allophycocyanin (APC) | 660 | Red |
|  | Allophycocyanin-Cyanine7 (APC-Cy7) | 785 | Infrared |
| 633 nm | Alexa Fluor 700 (AF-700) | 719 | Deep red |

**Table 4 | Overview of T cell subsets in BM, blood, and thymu****s (20-24 Months)**

| **Cell % of parent population** | | **C57BL/6 WT(n=10)** | | | | **GHR-/- (n=12)** | |  | |
| --- | --- | --- | --- | --- | --- | --- | --- | --- | --- |
|  | | **Mean SD** | | | | **Mean SD** | | **P value** | |
| **Spleen** | |  | | | |  | |  | |
| Leukocytes | | 8.151 | | | | 2.523 | | <0.0001 | |
| T cell | | 7.414 | | | | 3.064 | | 0.0840 | |
| Helper T cell | | 9.869 | | | | 9.793 | | 0.7437 | |
| Cytotoxic T cell | | 7.981 | | | | 12.61 | | 0.0384 | |
| Helper EM T cell | | 2.667 | | | | 7.696 | | <0.0001 | |
| Helper CM T cell | | 1.880 | | | | 6.864 | | 0.0001 | |
| Cytotoxic EM T cell | | 9.394 | | | | 4.771 | | 0.0008 | |
| Cytotoxic CM T cell | | 7.981 | | | | 12.61 | | 0.0002 | |
| Naïve 1 helper T cell | | 0.263 | | | | 0.609 | | 0.0303 | |
| Naïve 2 helper T cell | | 1.351 | | | | 1.224 | | 0.0072 | |
| Naïve 1 cytotoxic T cell | | 3.693 | | | | 12.76 | | <0.0001 | |
| Naïve 2 cytotoxic T cell | | 1.592 | | | | 4.408 | | 0.0072 | |
| **BM** | |  | | | |  | |  | |
| Leukocytes | | 2.210 | | | | 4.601 | | <0.0001 | |
| T cell | | 9.671 | | | | 4.354 | | <0.0001 | |
| Helper T cell | | 4.668 | | | | 7.287 | | 0.6972 | |
| Cytotoxic T cell | | 7.796 | | | | 7.365 | | 0.2287 | |
| Helper EM T cell | | 1.295 | | | | 5.236 | | 0.0002 | |
| Helper CM T cell | | 0.9555 | | | | 2.982 | | 0.0002 | |
| Cytotoxic EM T cell | | 5.122 | | | | 5.679 | | 0.0274 | |
| Cytotoxic CM T cell | | 5.049 | | | | 3.726 | | <0.0001 | |
| Naïve 1 helper T cell | | 0.2676 | | | | 1.442 | | 0.0542 | |
| Naïve 2 helper T cell | | 1.020 | | | | 1.141 | | <0.0001 | |
| Naïve 1 cytotoxic T cell | | 1.141 | | | | 6.454 | | <0.0001 | |
| Naïve 2 cytotoxic T cell | | 0.2076 | | | | 1.110 | | <0.0001 | |
| **Blood** | |  | | | |  | |  | |
| Leukocytes | | 6.524 | | | | 5.180 | | 0.0022 | |
| T cell | | 5.960 | | | | 4.620 | | 0.5502 | |
| Helper T cell | | 2.958 | | | | 3.646 | | <0.0001 | |
| Cytotoxic T cell | | 7.057 | | | | 6.610 | | 0.2093 | |
| Helper EM T cell | | 7.609 | | | | 13.37 | | 0.0177 | |
| Helper CM T cell | | 4.774 | | | | 6.381 | | 0.0687 | |
| Cytotoxic EM T cell | | 21.24 | | | | 15.63 | | 0.1938 | |
| Cytotoxic CM T cell | | 18.72 | | | | 6.687 | | 0.0472 | |
| Naïve 1 helper T cell | | 2.656 | | | | 3.989 | | 0.8913 | |
| Naïve 2 helper T cell | | 5.341 | | | | 6.043 | | 0.0078 | |
| Naïve 1 cytotoxic T cell | | 5.539 | | | | 16.77 | | 0.0074 | |
| Naïve 2 cytotoxic T cell | | 1.848 | | | | 5.125 | | 0.0002 | |
| **Thymus** | |  | | | |  | |  | |
| Leukocytes | | 25.43 | | | | 30.85 | | 0.54234 | |
| T cell | | 7.547 | | | | 7.974 | | 0.5046 | |
| SP helper T cell | | 7.099 | | | | 4.959 | | 0.3798 | |
| SP cytotoxic T cell | | | 1.462 | | | 3.081 | | | 0.2920 |
| DN T cell | | | 3.065 | | | 2.851 | | | 0.9948 |
| DP T cell | 11.63 | | | | 12.56 | | 0.8171 | | |
| Helper T cell | 11.43 | | | | 10.46 | | 0.7642 | | |
| Cytotoxic T cell | 8.988 | | | | 13.53 | | 0.4572 | | |
| Helper EM T cell | 18.45 | | | | 9.655 | | 0.4727 | | |
| Helper CM T cell | 0.3797 | | | | 1.277 | | 0.0306 | | |
| Cytotoxic EM T cell | 8.874 | | | | 5.463 | | 0.1798 | | |
| Cytotoxic CM T cell | 11.13 | | | | 5.519 | | 0.6932 | | |
| Naïve 1 helper T cell | 1.168 | | | | 1.277 | | 0.7681 | | |
| Naïve 2 helper T cell | 19.45 | | | | 10.23 | | 0.5764 | | |
| Naïve 1 cytotoxic T cell | 11.72 | | | | 15.65 | | 0.8690 | | |
| Naïve 2 cytotoxic T cell | 14.32 | | | | 13.94 | | 0.7548 | | |
| 20–24-month vs GHR-/-, n=10/12 | | | |  |  | |  | | |

| 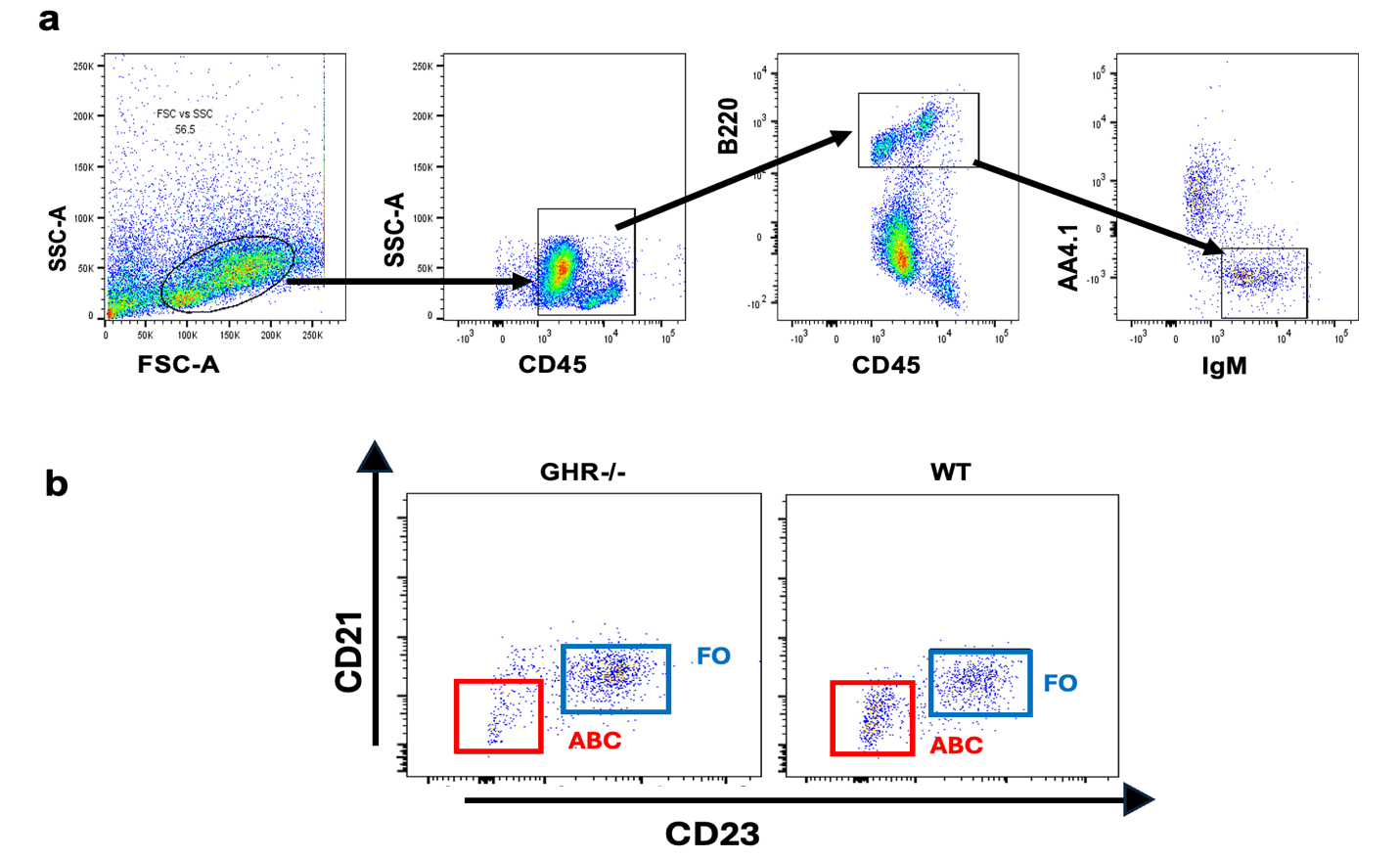**Supplemental Fig. 1** Composition of the BM B cell pool of old, age-matched GHR-/- and WT mice. The spleens of 21 to 24-month-old female GHR-/- and WT mice (n=6-7/genotype) were stained to evaluate percentages of the major B cell subsets by flow cytometry. (a) The gating strategy included sorting cells first by CD45+ and then by B220+ AA4.1-IgM+ cells to exclude transitional (AA4.1+) B cells. (b) A representative dot plot of splenic B cells from a single WT and GHR-/- spleen |
| --- |

**
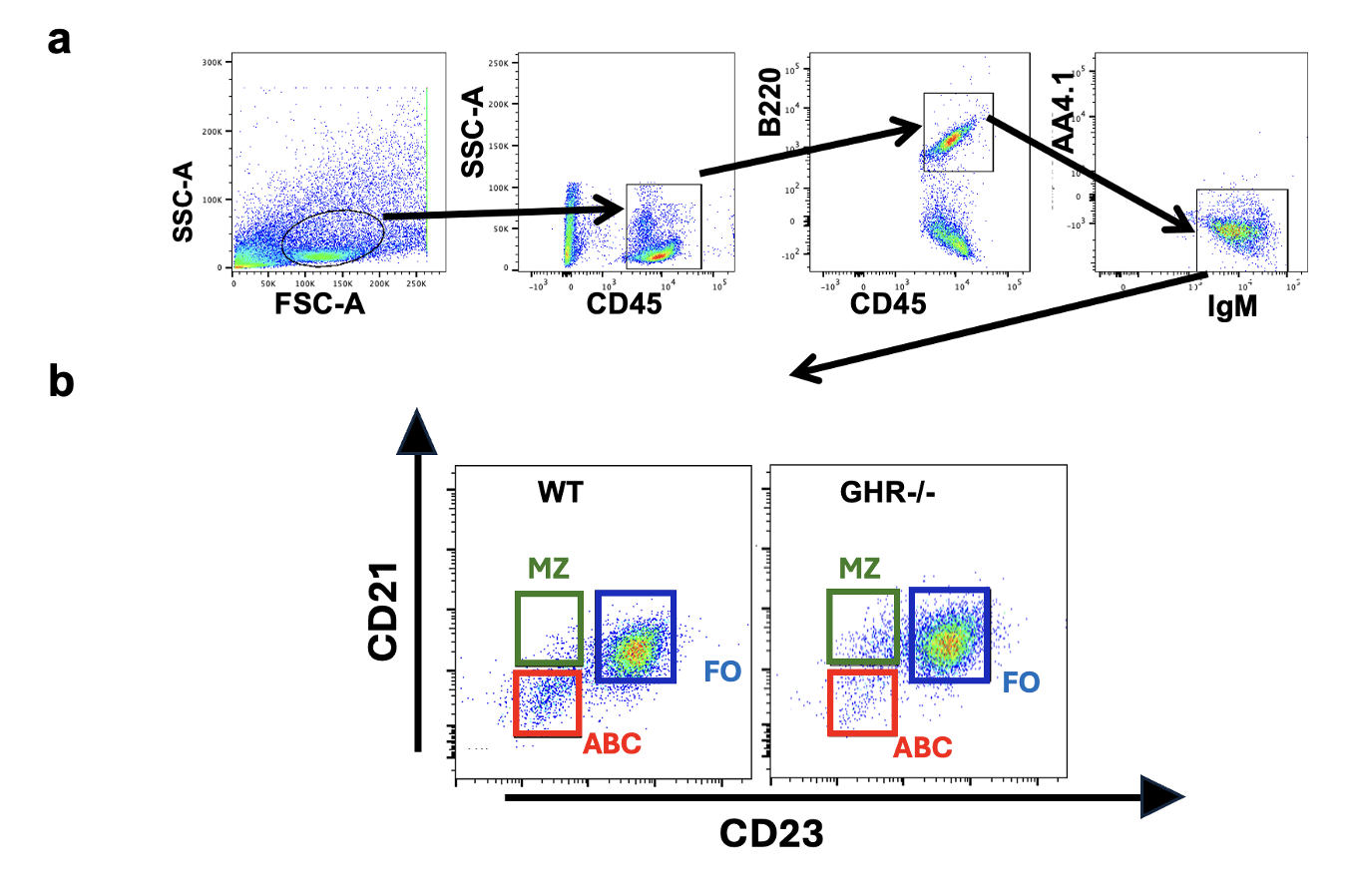
**

| **Supplemental Fig.2** Composition of the Blood B cell pool of old, age-matched GHR-/- and WT mice. The blood from 21 to 24-month-old female GHR-/- and WT mice (n=6-7/genotype) were stained to evaluate percentages of the major B cell subsets by flow cytometry. (a) The gating strategy included sorting cells first by CD45+ and then by B220+ AA4.1-IgM+ cells to exclude transitional (AA4.1+) B cells. (b) A representative dot plot of blood B cells from a single WT and GHR-/- spleen  **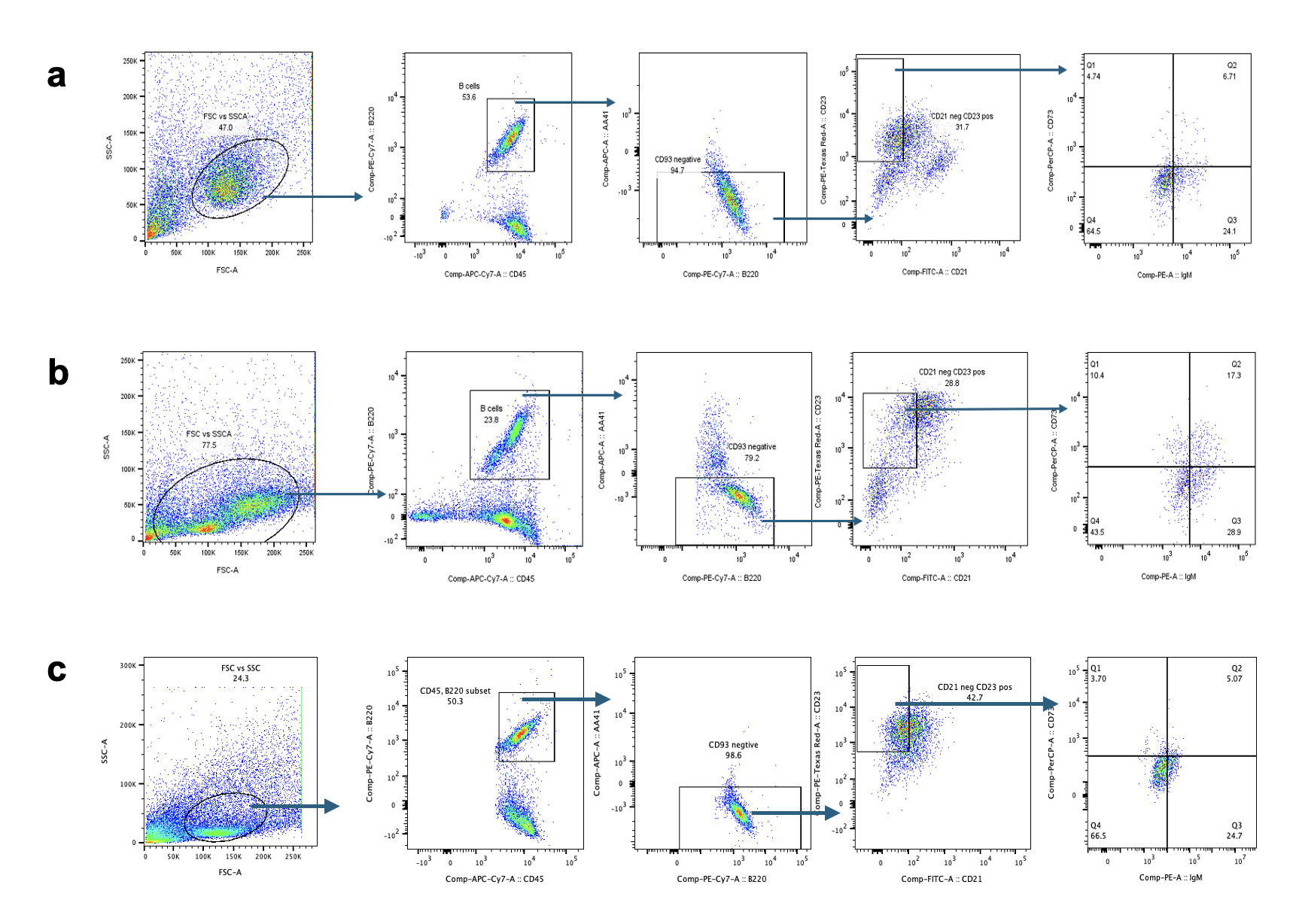** |
| --- |

| Supplemental Fig.3 Gating strategy for identification of memory B cells in a) spleen, b) bone marrow, and c) blood samples from female GHR -/- and WT mice at 21-24 month of age. To identify memory (CD45+ B220+ IgM+/-CD73+) B cells are first gated by CD45+(leukocytes), B220+(B cell), then by CD73+and as IgM+ and IgM- for identifying the unswitched and the switched populations. The same gating strategy was used for all tissue samples. |
| --- |

| 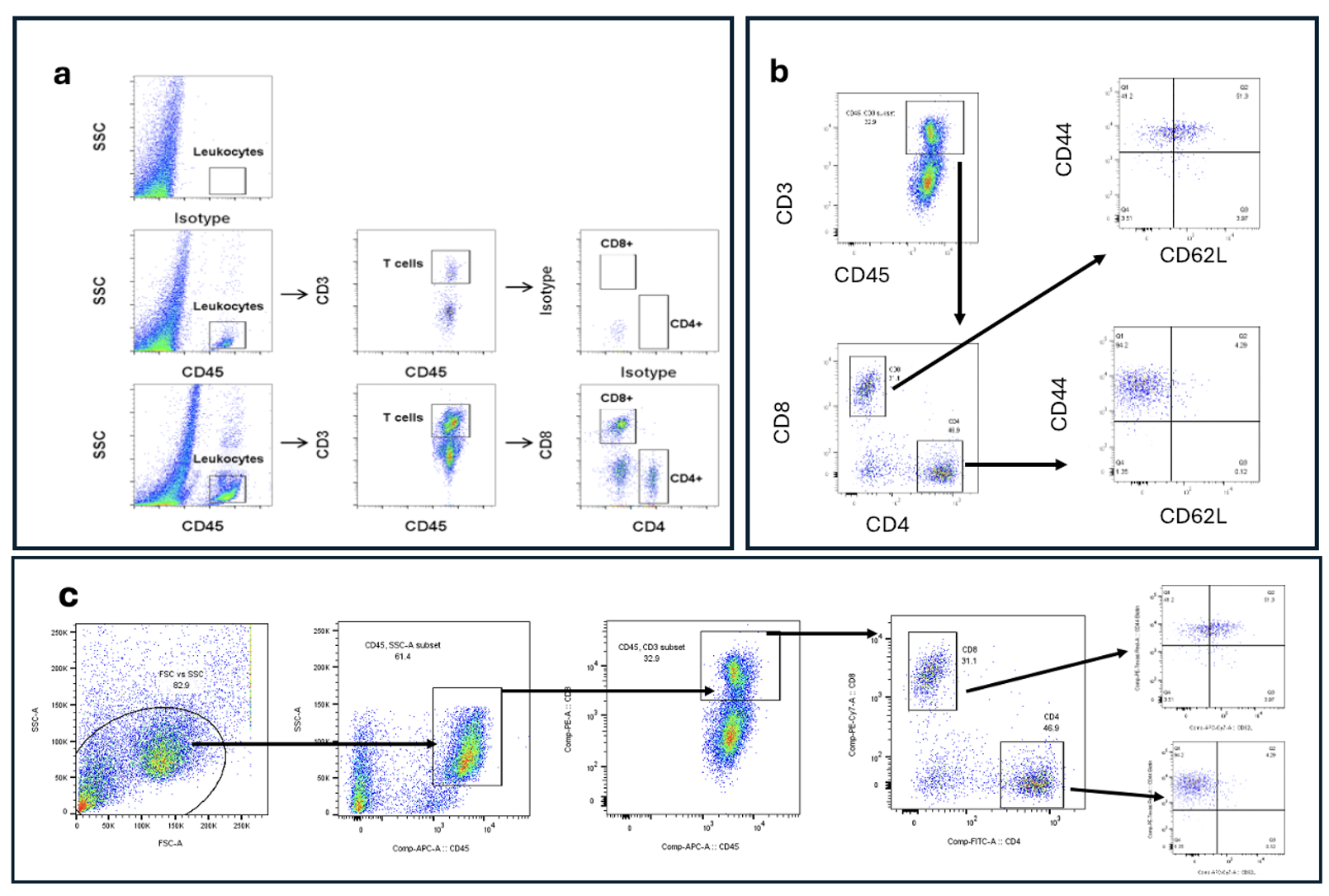 |
| --- |

**Supplemental Fig. 4** Composition of the spleen T cell pool of old, age-matched GHR-/- and WT mice. (a) Gating strategy was set for leukocytes (CD45+), T cells (CD45+CD3+), cytotoxic (CD8+) and helper (CD4+) T cells in spleen. (b) The memory cells were identified as CD44+. Subsequently, the CD44- cells were gated, after which CD62L+ cells were identified as naïve cells.

| 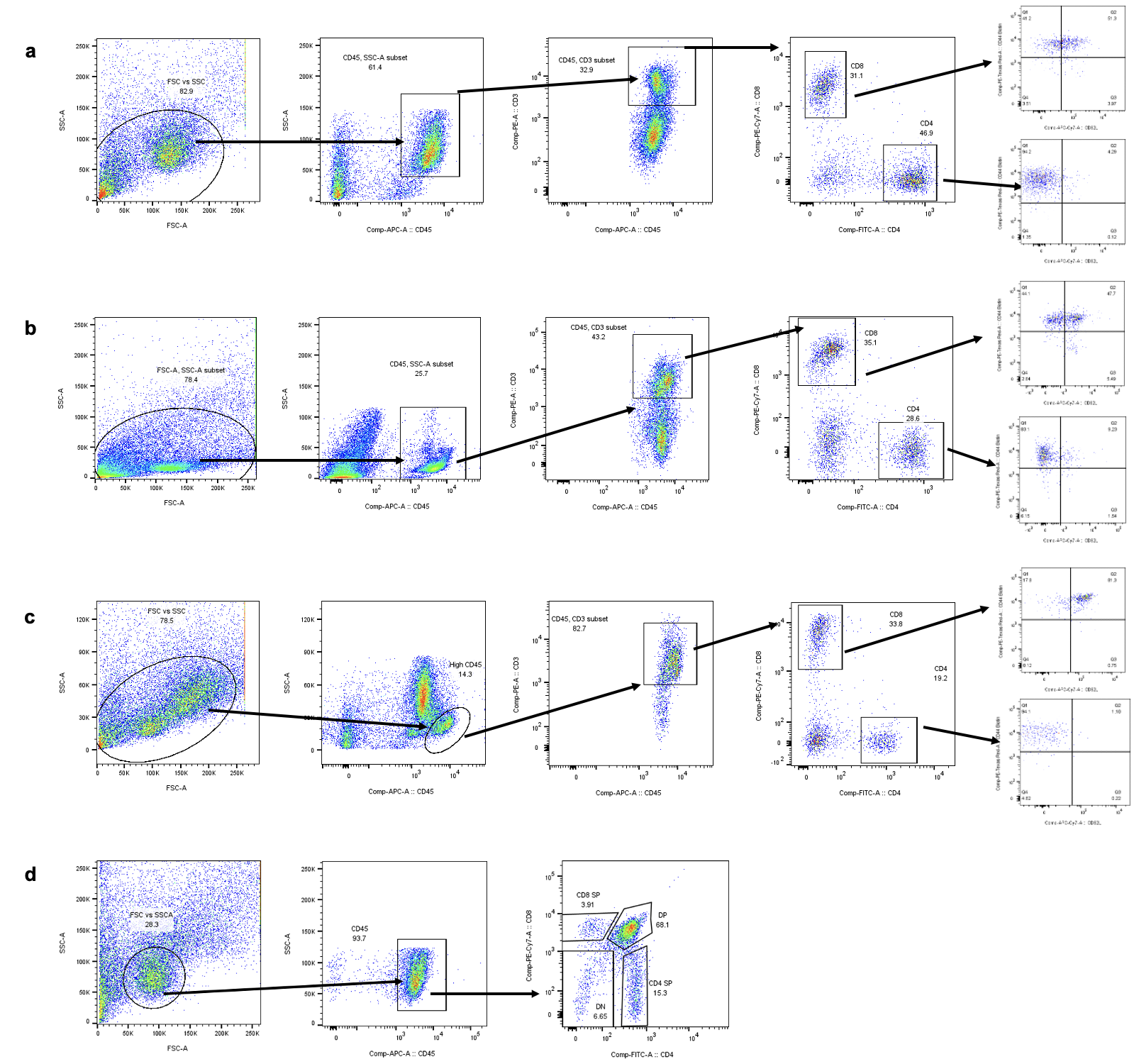 |
| --- |

**Supplemental Fig. 5** Composition of the T cell pool of old, age-matched GHR-/- and WT mice in spleen(a), BM(b), blood(c) and thymus(d). The tissues of 20 to 24-month-old female GHR-/- and WT mice (n=6-9/genotype) were stained to evaluate percentages of the major T cell subsets by flow cytometry. The gating strategy included gating cells first by CD45^+^ (Leukocytes), CD45^+^ CD3^+^ (T cells), CD4^+^ (Helper T cells), CD8^+^ (Cytotoxic T cells), CD4^+^ CD44^+^CD62L^-^ (EM; Effector memory helper T cells), CD4^+^ CD44^+^, CD62L^+^ (CM;CM; Central memory helper T cells), CD8^+^ CD44^+^CD62L^-^ (EM; Effector memory cytotoxic T cells ), CD8^+^ CD44^+^, CD62L^+^ (CM; Central memory cytotoxic T cells), CD4^+^CD44^-^CD62L^+^ (Naïve 1 helper T cells), CD4^+^CD44^-^CD62L^-^ (Naïve 2 helper T cells), CD8^+^CD44^-^CD62L^+^ (Naïve 1 cytotoxic T cells); and CD8^+^CD44^-^CD62L^-^ (Naïve 2 cytotoxic T cells). DP: double positive (CD4 and CD8 positive), DN: double negative (CD4 and CD8 negative), SP: single positive (CD4 or CD8), and DN are the most immature and SPs are mature T cells populations in thymus Fig. 5d

| 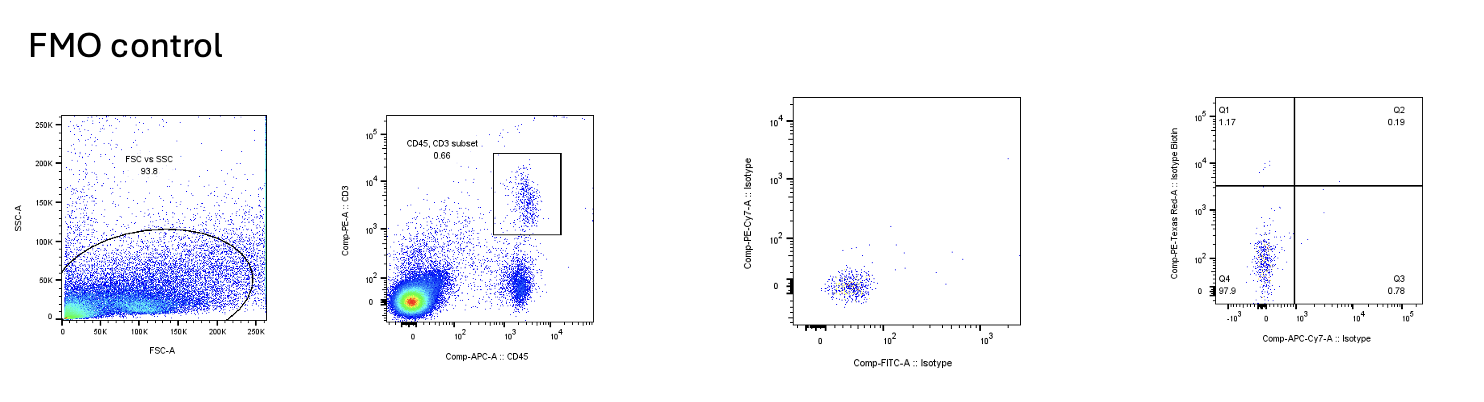 |
| --- |

**Supplemental Fig. 6** Representative florescence minus one control plots used to define positive gating thresholds for the indicated markers. This was performed to avoid fluorescence bleeding from the CD45 and CD3 channels when analyzing T cells. For that purpose, cells were stained with CD45 and CD3, and positive and negative boundaries for CD45 and CD3 were determined using corresponding isotype controls. Then, sample analysis, in addition to CD45 and CD3 isotype controls, we used this FMO control, with isotypes for the other fluorochromes to determine the boundaries for expression of different molecules within the CD45-CD3 gate. This provides better accuracy that only using isotype controls for all samples.
